# Maternal anemia and red blood cell requirements in 72 women undergoing *ex-utero* intrapartum treatment (EXIT) procedure

**DOI:** 10.3389/fmed.2024.1353405

**Published:** 2024-04-24

**Authors:** Jan Andreas Kloka, Thomas Jasny, Lukas Jennewein, Benjamin Friedrichson, Kai Zacharowski, Vanessa Neef

**Affiliations:** ^1^Goethe University Frankfurt, University Hospital, Department of Anaesthesiology, Intensive Care Medicine and Pain Therapy, Frankfurt, Germany; ^2^Goethe University Frankfurt, University Hospital, Department of Obstetrics and Perinatal Medicine, Frankfurt, Germany

**Keywords:** *ex-utero* intrapartum treatment, red blood cells, anemia, transfusion, pregnancy

## Abstract

**Background:**

The *ex-utero* intrapartum treatment (EXIT) allows to ensure fetal airway while keeping uteroplacental circulation. However, EXIT may become a life-threatening procedure due to the increased risk of uterine atony or placenta abruption with increased peripartum blood losses and increased transfusion rates. We aim to review maternal anemia prevalence and transfusion requirements in women undergoing EXIT procedure.

**Methods:**

Using data from the Federal German Statistical Office hospitalized women undergoing EXIT procedure between January 1st 2006 and December 31st 2021 were included. The prevalence of anemia, peripartum hemorrhage, comorbidities and administration of red blood cells (RBC) were analyzed.

**Results:**

In total, 72 women underwent EXIT procedure with a median age of 31 years (26;33.5). In 43.1% EXIT was conducted at 34–36 weeks of gestational age. “Anemia during pregnancy” was present in 47.2%, “anemia due to acute bleeding” in 25.0% and “iron deficiency anemia” in 15.3%. Postpartum hemorrhage occurred in 11.1%. RBCs were transfused in 15.3% of all women. Most women required 1–5 units of RBCs.

**Conclusion:**

Despite the rarity of this procedure, anemia management and blood conservation strategies in order to reduce the need for RBC transfusion are highly important in women undergoing EXIT procedure.

## Introduction

The *ex-utero* intrapartum treatment (EXIT) is used in neonates with upper airway obstruction with the aim to improve the infant‘s morbidity and mortality. During EXIT procedure, fetal airway can be established while uteroplacental circulation is still maintained (1).

In most cases the newborn is delivered via cesarean section when mothers are under deep general anesthesia (Sevoflurane 1.5–2%) with a potential high risk of uterine atony and postpartum hemorrhage (PPH). In Germany PPH is defined as blood loss >500 ml after vaginal delivery and >1,000 ml after cesarean section (2). There is large evidence, that PPH is a major risk factor for increased maternal morbidity and mortality (3, 4).

A comparative study by Noah et al. reveals, that peripartum blood losses (1,104 vs. 883 ml; *p* < 0.001 and postpartum complications (15.0 vs. 2.0%; *p* = 0.03) are significantly higher in women undergoing EXIT procedure compared to women with non-emergency cesarean section (5). Mostly based on case reports and case series, maternal blood loss during EXIT ranges from 400 to 2,000 ml (6–8). In a report of urgent EXIT procedure by Butwik et al. intrapartum blood loss was even higher (4,000 ml) with a total amount of seven transfused red blood cell (RBC) units during delivery (9).

The increased morbidity rate in women with severe hemorrhage is often aggravated by associated anemia during pregnancy (10). Anemia during pregnancy is a serious global health problem and affects about 42.0% of all pregnant women worldwide (11). Anemia during pregnancy is associated with impaired peripartum outcome and increased transfusion requirements (12). The main cause for anemia during pregnancy is iron deficiency causing iron deficiency anemia (IDA) due to increased iron demands (13).

Women undergoing EXIT procedure are at high risk for e.g., placental abruption or uterine atony resulting in anemia and increased transfusion requirements (14). Detection and treatment of pregnancy related anemia are strongly recommended in order to reduce the risk for RBC transfusion and decrease the mother's morbidity and mortality (15). Evidence in a large cohort of women undergoing EXIT procedure with focus on anemia and RBC transfusion rate is scarce. With the intention to improve maternal outcome, the present study aims to examine anemia prevalence, maternal hemorrhage and RBC transfusion requirements in women undergoing EXIT over the last 16 years in Germany, using a large database.

## Materials and methods

### Inclusion criteria

All hospitalized pregnant women between January 1st 2006 and December 31st 2021 in Germany undergoing EXIT procedure (*n* = 72) were included in the study.

### Availability of data and materials

In Germany, the reporting of diagnoses in hospitals is mandated by law to be carried out in accordance with the International Statistical Classification of Diseases and Related Health Problems (ICD) codes and the International Statistical Classification of Operation and Procedure Codes (OPS) (16). The data is stored at the local site of the German Federal Statistical Office. All calculations were conducted remotely, with the authors not having access to individual patient and hospital identifiers. According to §21 of the German Hospital Finance Law (KHG), all German hospitals are required to submit this data in anonymous form to the Institute for Hospital Remuneration (InEK) for further development of the DRG system. With use of anonymized data, the General Data Protection Regulation (GDPR) no longer applies. Consequently, informed consent from patients cannot be obtained due to anonymization. As the registry data had been anonymized to the authors, the need for ethical approval was waived by the Ethics Committee of the University Hospital Frankfurt (Chair: Prof. Dr. Harder, Ref: 2022-766).

In a recently published study data from the German Federal Statistical Office in >6 million pregnant women from 2011 to 2020 were analyzed. Briefly, anemia rate during pregnancy was 23.74% and RBC transfusion rate 1.23% (10). In the present study, focus was on anemia and RBC transfusion only in pregnant women undergoing EXIT procedure from 2006 to 2021.

### Definitions and data acquisition

Data from all age groups between 2006 and 2021 were analyzed. More recent data were not available due to accounting considerations and the internal data validation procedures of the Federal Statistical Office. The data is processed, its validity assessed, and i subsequently released for further scientific analysis.

Data include demographics (e.g., age), comorbidities (e.g., anemia), and complications (e.g., PPH). Diagnoses were coded in accordance with the 10th revision of the International Classification of Diseases, and procedures were coded following the International Classification of Procedures in Medicine. The assignment of OPS and ICD-10 codes to the corresponding procedures and diseases can be found in Supplementary Table 1.

### Definition of anemia and RBC transfusion

Anemia was defined according to the World Health Organization (WHO) with hemoglobin (Hb) concentration <11 g/dl in pregnant women (17). Regarding coding of anemia, there are different forms of anemia in ICD-10, which can be divided into general codes for anemia (that are not specific to pregnancy but may be coded in a case of a pregnant woman) and specific codes for anemia in obstetrics (marked with an “O” for obstetrics, see Supplementary Table 1). “Dietary anemia,” “Any other form of anemia,” “Anemia due to acute bleeding,” and “Iron deficiency anemia” are general codes, “anemia during pregnancy” is a code for any unspecified form of anemia during pregnancy. The codes cannot be added up, as multiple coding in one case is possible.

Transfusion of RBC was in accordance with the German transfusion guidelines. Briefly, RBC transfusion is recommended in asymptomatic patients with Hb <6 g/dl, in patients with cardiovascular risk factors with Hb between 6 and 8 g/dl or in patients with clinical symptoms of anemic hypoxia (18).

### Statistical analysis

Categorical variables are expressed as absolute numbers and percentages. Continuous variables were tested for normality. All considered continuous variables (age, hospital length of stay) were non-normally distributed. Hence, continuous variables were presented as the median with 25 and 75% quartiles. The statistical significance level was set to 5%. Excel 2019 (Microsoft Corp., Seattle, WA, USA) was used for data handling and SAS (Version 9.4M6, SAS Institute Inc., Cary, NC, USA) for statistical analysis.

## Results

In total, *n* = 72 pregnant hospitalized women undergoing EXIT procedure between January 1st 2006 and December 31st 2021 were included in analyses.

### Procedures in women undergoing EXIT procedure

In *n* = 72 women undergoing EXIT procedure “other obstetric surgeries” (including tamponade of the uterus or drainage of haematoma) were coded *n* = 102 (141.7%) times due to possible multiple coding of more than one “other obstetric procedure” in one case. In *n* = 7 (9.7%) “other operations for labor induction and child birth” were conducted. This is a comprehensive term and includes several operations and procedures. Of the *n* = 7 women reported, in *n* = 4 (5.6%) women “artificial rupture of the amniotic sac (amniotomy)” and in *n* = 3 (4.2%) women “operative measures on the fetus to facilitate labor” were coded. Additional coded procedures include magnetic resonance imaging prior to delivery in *n* = 15 (20.8%) of all women. In *n* = 4 (5.6%) arterial cannula was placed for EXIT procedure (see Table 1).

**Table 1:** Coded procedures and diagnoses of women undergoing *ex-utero* intrapartum treatment.

	**EXIT procedure**	
	***n***	**%**
Total	72	
**Diagnosis**		
Maternal care for (suspected) other fetal anomalies or impairments	20	27.78
Preterm spontaneous contractions with premature delivery	9	12.5
Maternal care due to hydrops fetalis	8	11.11
Preterm delivery without spontaneous contractions	8	11.11
Obstruction of labor due to other fetal anomalies	4	5.56
**Procedures**		
Other obstetric surgeries	102	141.67
Treatment during pregnancy	27	37.5
Magnetic resonance imaging	15	20.83
Monitoring of respiration, heart, and circulation	8	11.11
Other operations for labor induction and during childbirth	7	9.72
Therapeutic catheterization and vessel cannulation	4	5.56
Pain management	4	5.56

EXIT, *ex-utero* intrapartum treatment.

### Demographic characteristics of all women undergoing EXIT procedure

Median [Interquartile range (IQR)] age in all patients was 31 (26;33) years. In most women EXIT procedure was conducted at 34–36 weeks of gestational age (43.1%). Regarding existing pregnancy related comorbidities, only obesity (WHO grade I-III) was present in *n* = 3 (4.2%). Median (IQR) hospital length of stay was 185 [120;315] h (see Table 2).

**Table 2:** Characteristics of women undergoing *ex-utero* intrapartum treatment.

	**Patient characteristics**	
	***n***	**%**
Total patients undergoing EXIT procedure	72	
Age, median (Q1;Q3)	31 (26;33.5)	
Length of stay, hours (Q1;Q3)	185 (120:315)	
**Age groups**		
15–19	^*^	^*^
20–24	10	13.89
25–29	17	23.61
30–34	28	38.89
35–39	12	16.67
40–44	^*^	^*^
**Gestational week at delivery**		
26–33	23	31.94
34–36	31	43.06
37–41	18	25
**Comorbidities**		
Essential hypertension	0	0
Gestational hypertension	^*^	^*^
Diabetes during pregnancy (pre-existing)	0	0
Gestational diabetes	^*^	^*^
Nicotine abuse	0	0
Obesity	3	4.17
**Anemia**		
Vitamine B12-, folic acid-, any other dietary anemia	^*^	^*^
Any other form of anemia	21	29.17
Anemia due to acute bleeding	18	25
Anemia during pregnancy	34	47.22
Iron deficiency anemia	11	15.28
**Blood products**		
RBC	11	15.28
**Amount of administered RBCs**		
1–5	10	13.89
6–10	^*^	^*^
**Bleeding**		
Prepartum hemorrhage	0	0
Intrapartum hemorrhage	4	5.56
Postpartum hemorrhage	8	11.11

^*^ ≤ 3 cases are censored in each subcategory for privacy reasons. RBC, Red blood cell; EXIT, *ex-utero* intrapartum treatment.

### Anemia, peripartum hemorrhage and RBC transfusion on women undergoing EXIT procedure

Overall, the rate of “anemia during pregnancy” was present in *n* = 34 (47.2%), “any other form of anemia” in *n* = 21 (29.2%), “anemia due to acute bleeding” in *n* = 18 (25.0%) and IDA in *n* = 11 (15.3%). Bleeding complications, regarding intrapartum hemorrhage occurred in *n* = 4 (5.6%) and PPH in *n* = 8 (11.1%) of all women. Red blood cells were transfused in *n* = 11 (15.3%) of all women undergoing EXIT procedure. Most women required 1–5 units of RBCs (*n* = 10, 13.9%; see Table 2).

### EXIT procedure conducted over time in Germany

From 2010 to 2021 the amount of EXIT procedures performed in Germany ranged from *n* = 3 to 8 per year. In 2020 a maximum of *n* = 13 EXIT procedures were performed (see Table 3). Data reveals, that 75.0% of EXIT were performed at public hospitals with >1.000 beds. The remaining hospitals performing EXIT were censored due to the small number of cases of this rare procedure.

**Table 3:** *Ex-utero* intrapartum treatment over time.

**Year**	**EXIT procedure (*n*)**	**%**	**All deliveries (*n*)**	**%**
2006	^*^	^*^		
2007	^*^	^*^		
2008	^*^	^*^		
2009	^*^	^*^		
2010	4	5.56	656,390	0.000609
2011	6	8.33	642,791	0.000933
2012	5	6.94	572,448	0.000873
2013	3	4.17	579,948	0.000517
2014	3	4.17	613,993	0.000480
2015	3	4.17	633,665	0.000473
2016	4	5.56	679,095	0.000589
2017	7	9.72	682,094	0.001026
2018	4	5.56	682,745	0.000585
2019	8	11.11	674,645	0.001185
2020	13	18.06	676,041	0.001922
2021	5	6.94	698,932	0.000715

^*^ ≤ 3 cases are censored in each subcategory for privacy reasons. EXIT, *ex-utero* intrapartum treatment.

## Discussion

This retrospective study includes a cohort of 72 hospitalized women undergoing EXIT between January 2006 and December 2021 in Germany. “Anemia during pregnancy” was present in 47.2% of all women. Postpartum hemorrhage occurred in 11.1% and RBCs were administered in 15.3%.

In general, anemia during pregnancy is frequent (11). Our findings of an anemia prevalence of 47.2% in women undergoing EXIT procedure is higher compared to recently published studies. A nationwide analysis on anemia prevalence in Germany published in 2023 revealed an anemia rate of 23.7% in > 6 million pregnant women (10). Another recently published meta-analysis by Karami et al. including 52 studies involving 1,244,747 pregnant women revealed a global anemia prevalence of 36.8% [95% confidence interval [CI] 31.5–42.4]. Anemia was mostly present in the third trimester [48.8% [95% CI 38.7–58.9]] (19). As our study demonstrated, fetal delivery via EXIT is mostly conducted between 34 and 36 weeks of gestational age, when anemia rate is the highest. Since this is the first time investigating maternal anemia prevalence in a large cohort of women undergoing EXIT procedure, comparison with other studies on this procedure is not feasible. A small retrospective analysis of twelve medical records of women undergoing EXIT procedure reveals a mean postpartum Hb value after EXIT of 8.4 g/dl. (20).

Increased iron demands of ~1 g over the entire course of pregnancy often result in IDA (21, 22). In our study IDA was coded in 15.3% of all women. While it remains uncommon for pregnant women to be checked for ID unless anemic, a recent study indicates a prevalence of 42.0% of isolated ID (23). A study by Teichman et al. on 44,552 pregnant women demonstrates a screening rate for ID of 59.4%. Here, the majority of women were checked during the first trimester, when the risk of ID is lowest. Interestingly, in anemic women a subsequent test for ferritin was conducted only in 27.3% (24). As the diagnosis of IDA requires further measurement of iron parameters which are associated with increased financial expenses, it may be assumed, that IDA is undercoded and underdiagnosed in the present study. It is noteworthy, that the timing of antenatal anemia investigation and administration of intravenous iron is not mandatory for coding and therefore not available in the present study. However, national (e.g., National Institute for Health Care Excellence [NICE]) and international guidelines strongly recommend screening for hematological conditions with a full blood count at 28 weeks of gestation, as well as at any time during pregnancy if anemia is present (15, 25). The Network for the Advancement of Patient Blood Management, Haemostasis and Thrombosis (NATA) statement recommends the routine antenatal administration of oral iron (30–60 mg/day) and folic acid (400 μg/day). In the third trimester, antenatal intravenous iron should be administered in severe IDA (Hb <8 g/dl) or newly diagnosed IDA (15). To date, there is no explicit recommendation regarding anemia management in women undergoing EXIT. However, the high rate of “anemia during pregnancy” of 47.2% emphasizes the urgent need to implement this special patient population in national and international guidelines.

Interestingly, comorbidities were not present more often in women undergoing EXIT procedure compared to women undergoing vaginal or cesarean delivery. However, women undergoing EXIT procedure were older [31 [26;33.5] years] compared to pregnant women without the need for EXIT [28 [27.5;31] years] (10).

Overall, PPH increases the risk for RBC transfusions associated with potential maternal complications (15, 26, 27). In our analyses intrapartum hemorrhage occurred in 5.6% and PPH in 11.1% in all EXIT procedures conducted. These bleeding rates are higher compared to intrapartum hemorrhage (0.4%) and PPH (4.8%) in >6 million women undergoing vaginal delivery or cesarean delivery in Germany (10). RBC transfusion rate was 15.3% in all women. These findings are in line with case reports and case series on EXIT procedures published in literature. A study on 65 women undergoing EXIT procedures at the Children's Hospital of Philadelphia between 1998 and 2011 reports an RBC transfusion rate of 10.8% (seven out of 65 women). Two mothers required transfusion of 1 RBC unit, two mothers received 2 RBC units, two mothers were transfused with 3 RBC units, and one mother received 6 RBC units. Reasons for blood transfusion included placental abruption (*n* = 3), bleeding from uterine venous lakes after hysterotomy (*n* = 1), uterine atony (*n* = 1), preoperative anemia (*n* = 1), and postpartum anemia (*n* = 1). Mean intrapartum blood loss in transfused women was 1,500 ml (28). In another study on 45 women undergoing EXIT procedure median (IQR) maternal blood loss was 800 (500–2,000) ml; therefore 6 (13.3%) women received allogeneic blood transfusion (8). Emergent EXIT procedures are associated with even higher blood losses exceeding 4,000 ml (9).

Considering the high risk of increased maternal blood loss during EXIT procedure due to uterine atony or placental abruption (14), the need for blood conservation strategies to reduce intrapartum and PPH is high. There is proof, that intravenous iron reduces the need for RBC transfusions and constitutes an alternative to transfusion in profound IDA (29). Furthermore, cell salvage should be considered in obstetrics in women undergoing EXIT with anticipated high risk for severe hemorrhage or in case of unanticipated bleeding during surgery, along with other measures such as use of tranexamic acid (30).

## Limitations

One of the limitations in the present study is its retrospective nature and the utilization of secondary reimbursement data. Reimbursement data have a correlation with the medical cases in the hospital (31). However, it cannot be entirely precluded that certain conditions or events might be either over- or under-represented. Nonetheless, there exists an increased incentive for accurate documentation, as the medical service of the health insurance funds conducts audits on hospital reimbursements. It is noteworthy, that in the present study data on the severity of anemia and degree of bleeding in women undergoing EXIT procedure, the administration of intravenous iron and the reason for RBC transfusion are not available. Also, the number of women with both, intrapartum hemorrhage and PPH is not available. However, to improve patient care in especially this special patient population, these parameters should be considered in future studies, to improve the women‘s peripartum medical condition e.g., in case of anemia or the need for RBC transfusion. Alongside with the degree of bleeding and allogeneic RBC requirements, the medical urgency of EXIT procedure (emergency or elective with time for anemia correction) remains unclear with due to anonymization. As the focus of the present study is on the improvement of maternal peripartum care, fetal data (e.g., fetal reasons for EXIT, twins) are not available due to anonymization. The parameters chosen for this study were based on their high medical relevance, aiming to minimize the occurrence of coding errors. Data were collected in a structured and representative manner according to the Declaration of Helsinki. Laboratory findings or medication are not coded for reimbursement and are therefore not available for analysis. Last, this study uses reimbursement data for a health service research to improve patient care and to draw comprehensive conclusions. Individual patient data cannot be retrieved due to privacy reasons. Data such as e.g., placentation, gravida, parity, duration of anesthesia, and surgical time are not available to the authors. Since EXIT is a rare procedure in Germany, large prospective multicenter trials should include these variables in future investigations.

## Conclusions

The prevalence of anemia during pregnancy in women undergoing EXIT is high (47.2%). Intrapartum hemorrhage occurred in 5.6% and postpartum hemorrhage in 11.1% of all women. RBC transfusion rate was 15.3%. The aim of the present study is to increase the attention of this special and rare patient population regarding peripartum anemia management. So far, it is the largest analysis of women undergoing EXIT procedure with focus on anemia prevalence and administration of blood products. Despite the rarity of this procedure but existing risk for uterine atony and increased maternal blood loss, prepartum anemia management in women undergoing EXIT procedure has great potential to reduce the need for allogeneic blood transfusion. Still, there is a tremendous need for future research in order to improve maternal outcome in women undergoing EXIT.

## Data availability statement

The data on which the results of this study are based are available from the Federal Statistical Office with the restrictions applied. The dataset was used under license for the current study and is therefore not generally accessible. However, the data are available from the authors on reasonable request and with permission from the Federal Statistical Office. Requests to access these datasets should be directed to jan@dsgfrankfurt.de.

## Author contributions

JK: Writing – review & editing, Writing – original draft, Supervision, Project administration, Methodology, Formal analysis, Data curation, Conceptualization. TJ: Writing – review & editing, Methodology, Formal analysis, Data curation. BF: Writing – review & editing, Methodology, Formal analysis, Data curation, Conceptualization. KZ: Writing – review & editing, Supervision, Project administration, Conceptualization. LJ: Writing – review & editing, Conceptualization. VN: Writing – review & editing, Writing – original draft, Supervision, Project administration, Methodology, Formal analysis, Data curation, Conceptualization.
