# Supplementary material for: Maternal anemia and red blood cell requirements in 72 women undergoing ex-utero intrapartum treatment (EXIT) procedure
Source: Front Med (Lausanne). 2024 Apr 24;11:1353405. doi: 10.3389/fmed.2024.1353405 (PMC11076707; doi:10.3389/fmed.2024.1353405)
Supplement: Supplementary file 1 [file Table_1.docx]

**Supplemental Table 1: ICD-10 and OPS allocations**

| Diagnosis | OPS Codes | ICD-10 |
| --- | --- | --- |
|  |  |  |
| Essential hypertension |  | I10.- |
| Gestational hypertension |  | O13 |
| Diabetes during pregnancy |  | O24.- |
| Nicotine abuse |  | F17.2 |
| Obesity |  | E66.- |
| Vitamine B12-, folic acid-, any other dietary anemia |  | D51.x-D53.x |
| Any other form of anemia |  | D55.x - D64.x |
| Anemia due to acute bleeding |  | D62 |
| Anemia during pregnancy |  | O99.0 |
| Prepartum hemorrhage |  | O46.- |
| Intrapartum hemorrhage |  | O67.- |
| Postpartum hemorrhage |  | O72.- |
| RBC | 8-800.c |  |
| Maternal care for (suspected) other fetal anomalies or impairments |  | O35.8 |
| Preterm spontaneous contractions with premature delivery |  | O60.1 |
| Maternal care due to hydrops fetalis |  | O36.2 |
| Preterm delivery without spontaneous contractions |  | O60.3 |
| Obstruction of labor due to other fetal anomalies |  | O66.3 |
| Other obstetric surgeries | 5-759 |  |
| Treatment during pregnancy | 9-280 |  |
| Magnetic resonance imaging | 3-80b |  |
| Monitoring of respiration, heart, and circulation | 8-930 |  |
| Other operations for labor induction and during childbirth | 5-734 |  |
| Therapeutic catheterization and vessel cannulation | 8-830 |  |
| Pain management | 8-910 |  |

RBC, red blood cell; OPS, International Statistical Classification of Operation and Procedure Codes; ICD, International Statistical Classification of Diseases and related Health Problems
